# Case Report: Clonal evolution of diffuse large B-cell lymphoma to plasmablastic lymphoma: diagnostic challenges in a case of gastric lesion with EBV-negative PBL

**DOI:** 10.3389/fonc.2026.1784860

**Published:** 2026-03-02

**Authors:** Jie Xu, Yueli Liu, Xi Feng, Dejie Zhao, Kui Liu, Siyuan Cui, Yan Wang

**Affiliations:** 1 Department of Hematology, Affiliated Hospital of Shandong University of Traditional Chinese Medicine, Jinan, China; 2 Department of Pathology, Affiliated Hospital of Shandong University of Traditional Chinese Medicine, Jinan, China; 3 Department of Vascular Surgery, Affiliated Hospital of Shandong University of Traditional Chinese Medicine, Jinan, China

**Keywords:** clonal relationship, composite lymphoma, diffuse large B-cell lymphoma, evolution, plasmablastic lymphoma

## Abstract

**Background:**

The clonal evolution from a diffuse large B-cell lymphoma (DLBCL) to a plasmablastic lymphoma (PBL) is uncommon, presenting remarkable clinical challenges. This phenomenon has critical diagnostic and therapeutic implications, particularly in cases of EBV-negative lesions that show immunophenotypic divergence.

**Case summary:**

We herein report an unusual case of two different immunophenotypes, namely DLBCL in the porta hepatis and concomitant PBL in the stomach. Immunoglobulin gene rearrangement analysis confirmed that the two tumors had the same clonal origin. The patient presented with characteristic PBL features, including loss of CD20 expression, high MYC expression, co-expression of BCL2, and a distinctive clinical manifestation involving gastric mucosa. The patient demonstrated only a transient response to initial therapy with R-CHOP plus bortezomib, following which she had rapid disease progression, resulting in an overall survival of 10 months.

**Conclusion:**

For extranodal lymphomas, multisite sampling should be performed for a confirmed diagnosis to prevent misdiagnosis or oversight of concurrent lesions. This diagnostically challenging case highlights the importance of molecular testing for identifying and understanding the clonal evolution of lymphomas and suggests how immunophenotypic heterogeneity may lead to misdiagnosis. It also provides important insights into the biology of DLBCL-to-PBL evolution. These findings highlight the need for more precise molecular diagnostic tools and novel approaches to improve outcomes for patients with such highly aggressive lymphoma.

## 1 Introduction

Diffuse large B-cell lymphoma (DLBCL) is the most common non-Hodgkin B-cell lymphoma with a very high genetic and phenotypic heterogeneity. This heterogeneity leads to variable responses to standard immunochemotherapy (1). Despite the curative potential of frontline chemotherapy, many patients experience a refractory or relapsing disease (2). Plasmablastic lymphoma (PBL) is one of the rarest and aggressive variants that may emerge and is often associated with immunosuppression and Epstein-Barr virus (EBV) infection, although EBV-negative cases rarely occur in immunocompetent hosts (3). PBL exhibits an immunophenotype of terminally differentiated B-cells without traditional B-cell markers (e.g., CD20) and brings great diagnostic challenges because of its overlapping characteristics with those of other aggressive B-cell and plasma-cell neoplasms (4).

In clinical practice, cases of concurrent occurrence of two types of lymphomas are observed, which highlights the intratumoral heterogeneity of lymphomas. This phenomenon is typically manifested in three ways (5). The first is the lymphoma transformation, characterized by the transformation of low-grade lymphoma into high-grade lymphoma during disease progression, with Richter transformation being the most prevalent one in chronic lymphocytic leukemia/small lymphocytic lymphoma. The second is the discordant lymphoma, characterized by the presence of at least two distinct histological subtypes at different sites. Histologic discordance most commonly occurs when lymph node biopsy shows an aggressive lymphoma while bone marrow biopsy indicates an indolent lymphoma. The third is the composite lymphoma, where two distinct types of lymphomas coexist within the same organ without any clonal relationship. The coexistence of these lesions with varying morphologic or immunophenotypic features in a single patient poses fundamental diagnostic challenges, suggesting either genuine transformation from common progenitor cells or the development of an independent second primary malignancy (6, 7). Hence, molecular clonality assessment, particularly immunoglobulin gene rearrangement analysis, plays a pivotal role in resolving diagnostic dilemmas and devising appropriate treatment plans (8).

This study presents the case of a patient who had non–germinal center B-cell–like (non-GCB) DLBCL and a concomitant gastric lesion with EBV-negative PBL. The discrepant immunophenotypes between the hilar lymph node DLBCL (CD20+) and the gastric PBL (CD20−) posed a substantial diagnostic challenge, raising the possibility of two distinct lymphoproliferative processes. Following the confirmation of clonal relationships through immunoglobulin gene rearrangement analysis, this case represents a rare instance of the transformation evolution of DLBCL into PBL in individuals with normal immune function. The coexistence of these two types of lymphoma differs from the heterogeneous manifestations observed in the aforementioned three lymphoma categories. This case underscores the clonal evolution present among aggressive lymphomas. Despite undergoing multi-drug combination chemotherapy, the patient exhibited rapid disease progression, underscoring the aggressive biological characteristics and intrinsic drug resistance associated with this lymphoma subtype.

## 2 Case description

**Figure 1:**
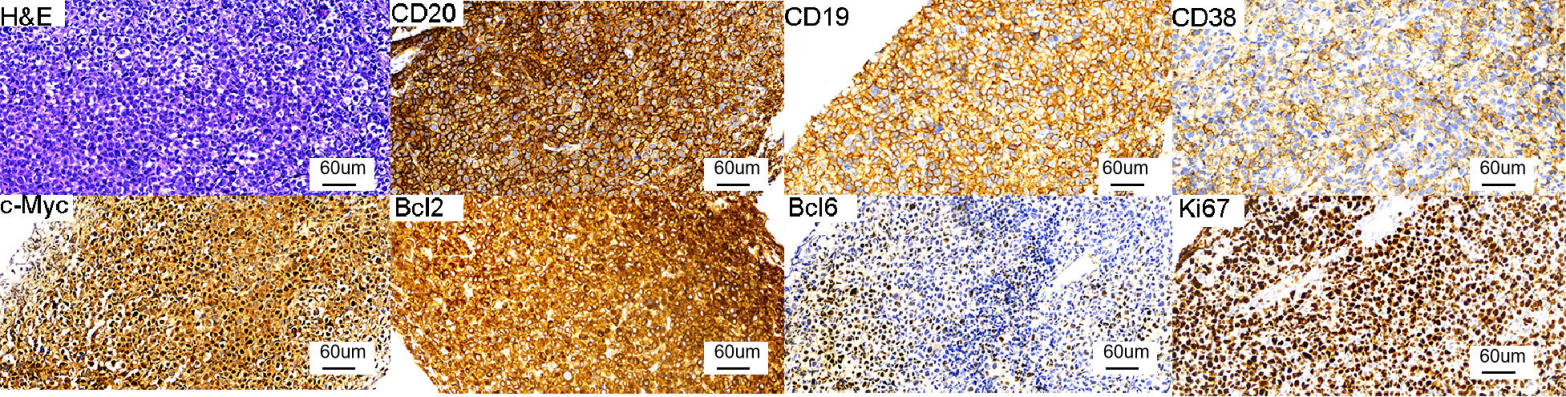
Histologic and immunohistochemical features of the hepatic hilar lymph node. Immunohistochemistry: CD20 (+), CD19 (+), CD38 (partially positive), C-MYC (60% positive), BCL2 (>90% positive), BCL6 (+), Ki-67 (80% positive). (all images at ×20 magnification).

A 46-year-old woman was hospitalized for recurrent fever and progressive fatigue. She had no previous history of chronic diseases. She received anti-infective therapy, but it failed to control her fever, and metagenomic pathogen test showed negative results. Her fatigue worsened, and laboratory tests revealed a hemoglobin level of 60 g/L. A previous bone marrow test revealed microcytic hypochromic anemia. Contrast-enhanced computed tomography (CT) of the chest and abdomen showed many enlarged lymph nodes in the hepatic hilum, lesser omental sac, mesenteric region, peritoneal and retroperitoneal spaces, and around the iliac vessels. Ultrasound-guided core needle biopsy was performed from a hepatic hilar lymph node of 5 cm diameter. Histopathology revealed DLBCL of the non–germinal center B-cell type. Immunohistochemistry revealed the following findings: BCL2 (>90% positive), BCL6 (+), CD3 (−), CD5 (−), CD10 (−), CD19 (+), CD20 (+), CD21 (−), CD79a (+), C-MYC (60% positive), CKpan (−), MUM1 (+), vimentin (+), CD22 (5% positive), CD38 (partially positive), cyclin D1 (−), CD30 (<1% positive), and Ki-67 (80% positive). *In situ* hybridization: EBER (−). (**Figure 1**).

**Figure 2:**
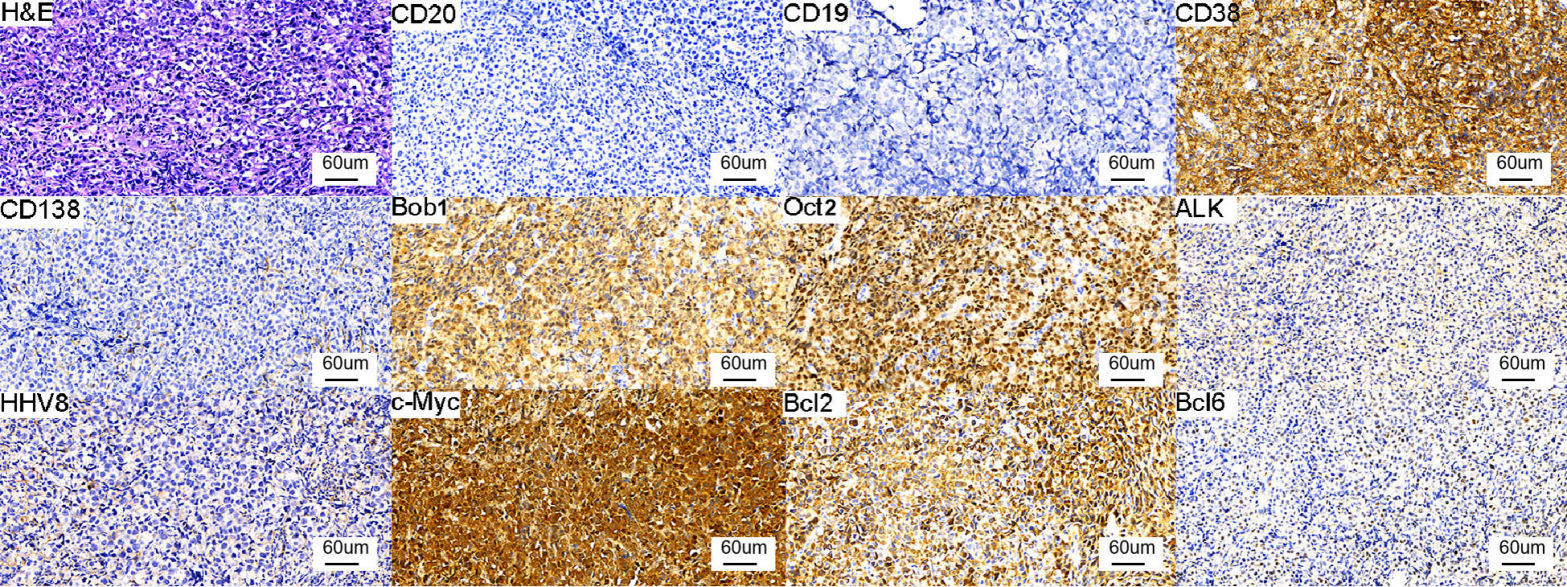
Histologic and immunohistochemical features of the gastric lesion. Immunohistochemistry: CD20 (−), CD19 (−), CD38 (+), CD138 (−), Bob1 (+), OCT2 (+), ALK (−), HHV8 (−), C-MYC (80% positive), BCL2 (+), BCL6 (weakly positive). (all images at ×20 magnification).

The patient presented with severe anemia. Bone marrow analysis revealed the absence of tumor cells, and no hemolysis was observed. The patient had no melena but showed a positive result in the fecal occult blood test. CT findings suggested gastric wall thickening, and endoscopy revealed a gastric space–occupying lesion at the junction of the gastric antrum and gastric body. Histopathology of the gastric biopsy showed findings consistent with PBL. Immunohistochemistry revealed the following findings: CD3 (−), CD5 (−), CD19 (−), CD20 (−), anaplastic lymphoma kinase (ALK) (−), BCL2 (+), BCL6 (weakly positive), CD10 (−), CD21 (−), CD30 (−), CD38 (+), CD45 (+), CD79a (partially positive), CD138 (−), human herpesvirus 8 (HHV8) (−), cyclin D1 (−), C-MYC (80% positive), CKpan (−), Ki-67 (80% positive), MUM1 (+), PAX5 (partially weakly positive), OCT2 (+), and BOB1 (+). *In situ* hybridization showed EBER (−). (**Figure 2**).

Pathological specimens from the hepatic hilum and the gastric mass showed a similar tumor cell morphology. However, they exhibited distinct immunophenotypes, notably in terms of CD19, CD20, and CD38 expression. Two possibilities were therefore considered: a single tumor with regional heterogeneity or two distinct tumor types.

**Figure 3:**
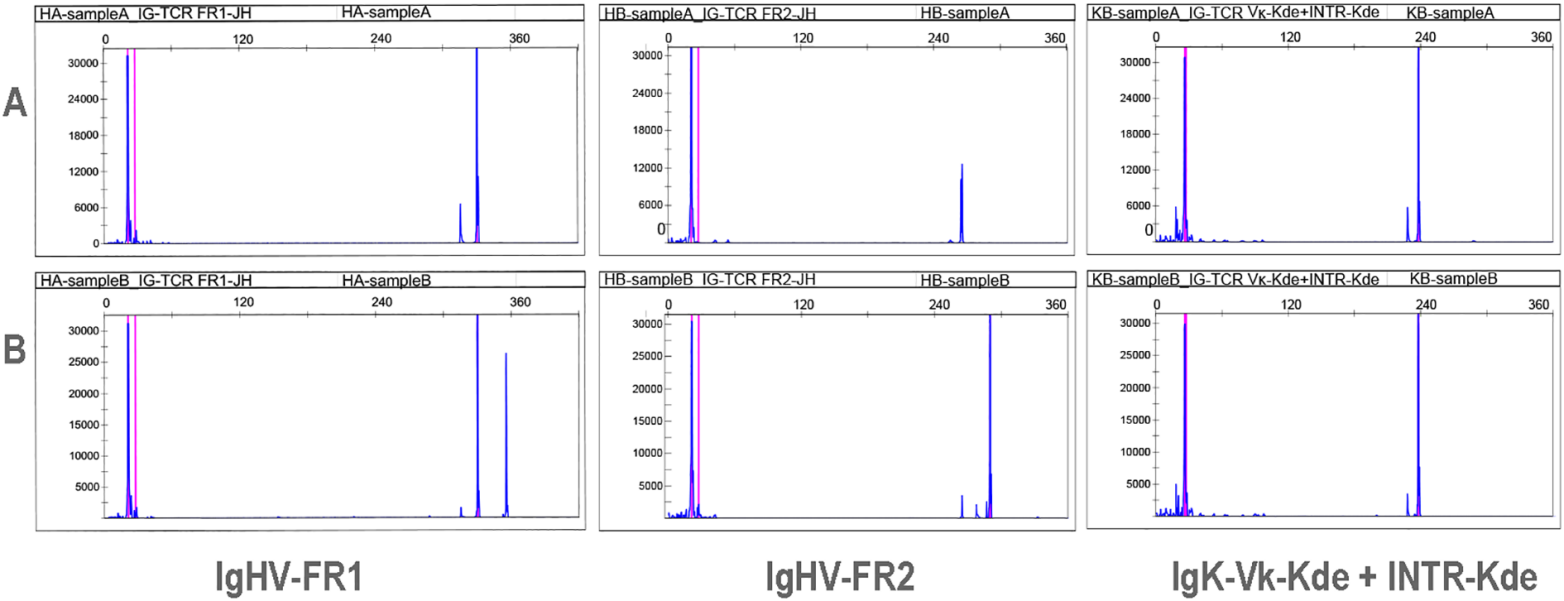
Capillary electrophoretic profiles of IG gene rearrangement in both samples. Sample **(A)** is the lymph node biopsy from the hepatic hilum, and sample **(B)** is the gastric biopsy. The results indicate that the clonal rearrangements of the IG genes in the two tissue samples are consistent.

Therefore, immunoglobulin gene rearrangement analysis was performed on both pathological specimens. DNA was extracted from paraffin-embedded tissues, and the extracted DNA was amplified using the polymerase chain reaction (PCR) assay and analyzed by capillary electrophoresis. The two specimens yielded concordant B-cell clonality profiles: IgHV-FR1 (+), IgHV-FR2 (+), IgHV-FR3 (−), IgK-Vk-Jk (−), and IgK-Vk-Kde (+) INTR-Kde (+; **Figure 3**). These findings indicate homology between the two tumors and support a diagnosis of evolution from DLBCL to PBL in the gastric lesion.

Accordingly, a diagnosis of DLBCL was made, along with a clonal evolution from DLBCL into PBL in the gastric region. Subsequently, she received rituximab, cyclophosphamide, doxorubicin, vincristine, and methylprednisolone (R-CHOP) combined with bortezomib. She was anemia free after 2 cycles. By 4 cycles, contrast-enhanced CT findings showed reduced intra-abdominal and retroperitoneal lymph nodes compared with baseline, suggesting partial response. Following an additional 4 cycles of treatment, a notable soft tissue density lesion was identified at the hepatic hilum (axial diameter of approximately 11 cm) with focal gastric wall thickening and local soft tissue mass, suggesting disease progression. The treatment was then switched to zanubrutinib plus gemcitabine and oxaliplatin (GemOx). After another 2 cycles, a repeat CT scan showed no reduction in the hepatic hilum lymph node, and the disease progressed again. She died of infection, and the overall survival was 10 months (**Table 1**).

**Table 1:** Clinical timeline.

Date	Fundings/Interventions	Results
September 02, 2023	She was hospitalized for fever and fatigue	
September 05, 2023	CT findings showed many enlarged lymph nodes. Biopsy was performed from a hepatic hilar lymph node	Histopathology of the hepatic hilar lymph node was DLBCL
September 07, 2023	She was anemia free, and fecal occult blood test results were positive. A CT scan revealed gastric wall thickening, and endoscopy findings revealed a gastric space–occupying lesion	Histopathology of the gastric biopsy was PBL
September 20, 2023	Immunoglobulin gene rearrangement analysis was performed on both pathological specimens	The two specimens yielded concordant B-cell clonality profiles
September-November 2023	R-CHOP combined with bortezomib × 4 cycles	Partial response
December 2023-February 2024	R-CHOP combined with bortezomib × 4 cycles	Progressive disease
March 2024-April 2024	Zanubrutinib plus GemOx × 2 cycles	Continued disease progression
June 2024	Died of infection	Overall survival was 10 months

## 3 Discussion

**Figure 4:**
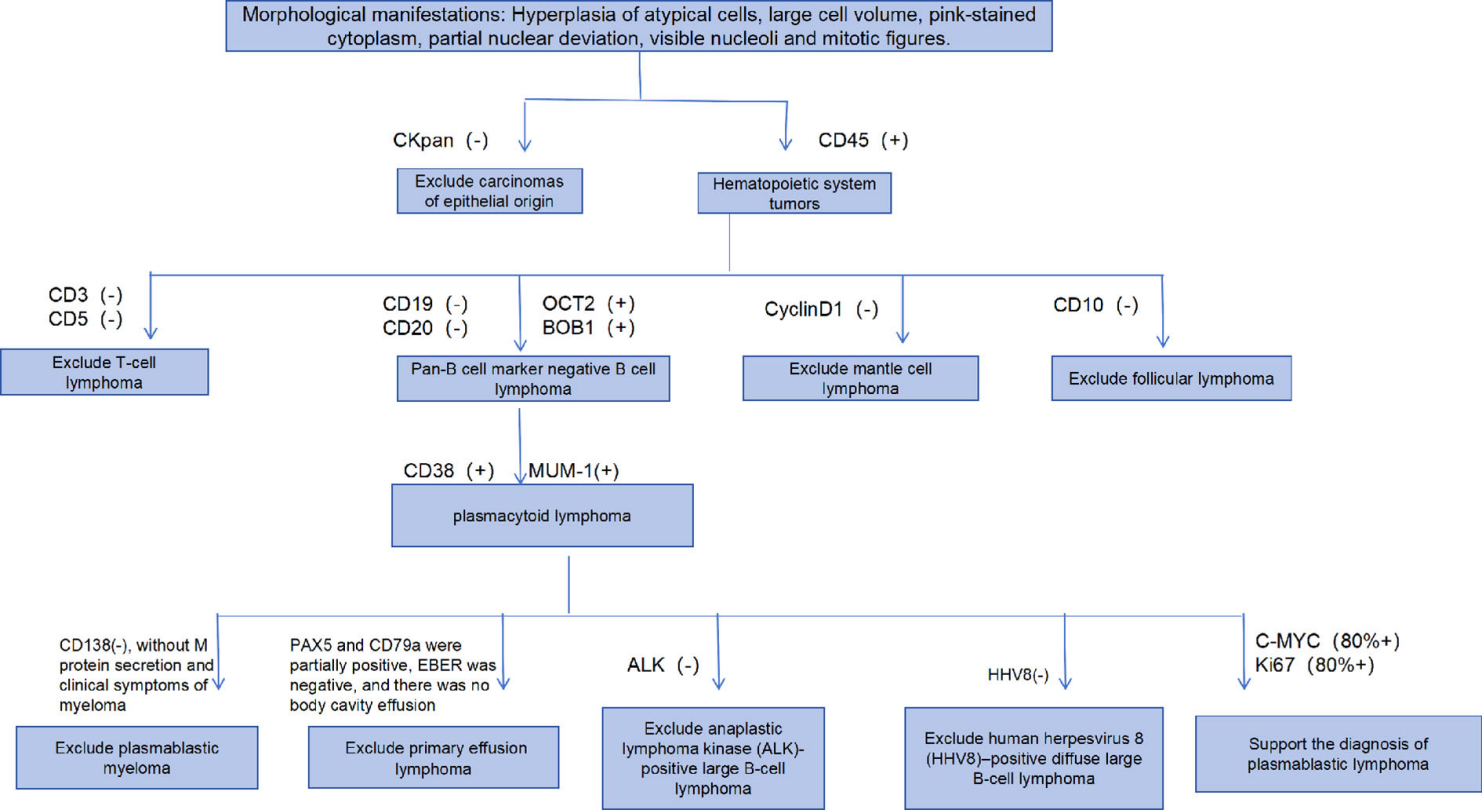
Process for the pathologic diagnosis of gastric lesions.

We herein report a case of concurrent DLBCL and extralymphatic PBL. A lymph node biopsy from the hepatic hilum showed the prototypical double-expressor DLBCL, whereas gastric biopsy showed loss of the canonical pan-B-cell markers CD19 and CD20 and presented morphological features of plasma-cell differentiation (9). Immunohistochemistry with OCT2 and BOB1 showed that the gastric specimen was obtained from a B-cell lineage despite the absence of pan-B-cell markers. The differential diagnosis for the gastric lesion with plasmablastic morphology included plasmablastic myeloma, primary effusion lymphoma, ALK-positive large B-cell lymphoma, and HHV8-positive DLBCL (10). The final diagnosis of plasmoblastic lymphoma of the stomach was clearly made (**Figure 4**). PCR assay of the immunoglobulin gene rearrangement on both gastric and nodal samples showed a common clonal origin of both lymphomas. The case was labeled as DLBCL together with evolution to gastric PBL. The PBL in this case was not CD138-negative (11). CD138-negative PBLs often occur at nonoral sites, have a weak association with EBV, and pose remarkable diagnostic challenges.

Such a case is exceptionally rare. Three cases have been reported previously (12–14), and their details are summarized in **Table 2**. Through the analysis of these cases, multisite biopsy is suggested as important for aggressive lymphomas with heterogeneous features. For lymphomas with extranodal lesions, even if a diagnosis has been made through lymph node biopsy, performing a biopsy of the extranodal lesions is still necessary to verify whether the results are consistent. Routine endoscopy is indicated when gastrointestinal or other hollow-organ involvement is suspected. If chemotherapy is ineffective or the disease progresses, biopsy specimens from clinically significant lesions should also be actively collected for re-evaluation.

**Table 2:** Summary of similar case reports.

Study	Imuta et al. (12)	Hashimoto et al. (13)	Carolina et al. (14)	Present case
Age(years)/Sex	84/F	37/M	55/F	46/F
Main symptoms	Chronic diarrhea	Swelling at the root of the nose	Thrombosis caused by a pelvic mass compressing the iliac vessels	Fever
Additional symptoms	/	Frequent urination	Thigh skin infiltration (after 2 cycles of chemotherapy)	Severe anemia and abnormal fecal examination
Involved lymph node regions	Not involved	Both the upper and lower sides of the diaphragm	Abdomen and mediastinum	Abdomen and pelvis
Extranodal lesions	From the cecum to the appendix vermiformis as well as the rectal tumor	Nasal mucosa, bilateral tonsils, prostate, and urinary bladder	Pelvic mass, thigh skin (after 2 cycles of chemotherapy)	Gastric mass
Biopsy site of DLBCL	Ileocecal lesion	Nasal mucosa	Pelvic mass	Hepatic hilar lymph node
Biopsy site of PBL	Rectal lesion	Urinary bladder	Thigh skin	Gastric mass
Homology between 2 lymphomas	Yes	Yes	No detection	Yes
Treatment	RCHOP	RCHOP	RCHOP/ESHAP	RCHOP plus bortezomib/zanubrutinib plus GemOx
Outcome	No recurrence for 1 year after the surgery	Recurrence of DLBCL after 4 years	Died 3.5 months after the diagnosis of plasmablastic transformation	Died 10 months after the diagnosis

DLBCL, diffuse large B-cell lymphoma; ESHAP, etoposide, cisplatin, methylprednisolone, and cytarabine; GemOx, gemcitabine and oxaliplatin; PBL, plasmablastic lymphoma; RCHOP, rituximab, cyclophosphamide, doxorubicin, vincristine, and methylprednisolone.

The molecular mechanisms driving the evolution from DLBCL to PBL include dysregulation of MYC and changes in the PRDM1/BLIMP1 pathway. In ABC-DLBCL studies (15), animal experiments have shown that NF-κB directly induces the expression of genes required for B-cell differentiation into plasma cells and that dysregulation of NF-κB activation and MYC overexpression also leads to B-cell conversion to the plasmablastoid phenotype. Inactivation of PRDM1 prevents B-cell differentiation to plasma cells (16). PBL often contains mutations of PRDM1 that affect its DNA-binding zinc finger domains, leading to loss of protein function, failure to regulate B-cell gene programs, and failure to fully initiate mature plasma-cell differentiation. This results in a “differentiation block” with the plasmablastoid phenotype. MYC abnormalities in PBL include translocations (e.g., t (8, 14)) and amplifications. This disorder dysregulates proliferative and apoptotic pathways and can cooperate with PRDM1 defects to arrest cells at the plasmablast stage. Co-expression of the aberrant proteins MYC and PRDM1 is a key mechanism for the PBL immunophenotype (17). In this case, gastric PBL was more abundantly expressed than DLBCL, suggesting that MYC upregulation could have been the driving factor for the evolution of DLBCL to PBL. However, these mechanistic interpretations must be regarded as provisional. Although clonal relatedness was demonstrated by PCR-based immunoglobulin gene rearrangement testing, comprehensive genomic profiling such as whole-exome sequencing (WES), bulk RNA sequencing, or single-cell transcriptomics was not performed. Consequently, the precise mutational landscape, clonal phylogeny, and pathway-level disturbances (including definitive alterations in MYC, PRDM1, or NF-κB pathway genes) remain undefined. Future studies should incorporate WES and single-cell RNA sequencing to delineate driver events, clonal architecture, and tumor microenvironment interactions that underlie the DLBCL-to-plasmablastic phenotypic evolution.

Many studies have shown that patients with EBV-positive DLBCL generally have poorer outcomes than those with EBV-negative DLBCL, and their overall survival and progression-free survival are significantly lower (18). PBL occurs most often in patients with human immunodeficiency virus (HIV) infection; moreover, PBL cells can be infected by EBV, and in HIV-positive cases, the infection rate is more than 70% (19). Regarding prognosis, a large international multicenter retrospective study of 281 patients with PBL showed that EBV-negative lymphoma was significantly associated with poor overall survival and that HIV status had no effect on survival (20). In the present case, the patient was negative for both HIV and EBV, suggesting that lymphoma arises in an immunocompetent host. Additionally, the DLBCL-to-PBL evolution occurred in a gastric mucosa lymphoid tissue microenvironment, suggesting a pathogen distinct from the classical EBV-driven pathway. The poor prognosis is consistent with published data on EBV-negative PBL.

The treatment of this case was challenged by concurrent double-expressor DLBCL of non-GCB subtype and clonally related plasmablastic lymphoma with CD20 loss. Initial therapy with R-CHOP combined with bortezomib was chosen to target NF-κB pathway activation, which is characteristic of activated B-cell-like (ABC) DLBCL (16), to overcome BCL2-mediated resistance commonly observed in double-expressor lymphomas (1), and to leverage bortezomib’s known activity against plasmablastic tumors through induction of endoplasmic reticulum stress (17, 20). Although a transient clinical response was achieved, disease progression subsequently emerged, primarily at the hepatic hilar mass, which had initially presented as CD20-positive DLBCL. Salvage therapy with zanubrutinib plus GemOx was subsequently administered, targeting this relapsed DLBCL component. This decision was informed by the non-GCB immunophenotype, which suggests dependence on B-cell receptor (BCR) and NF-κB signaling (21), preclinical evidence supporting synergistic effects between Bruton’s tyrosine kinase (BTK) inhibitors and DNA-damaging chemotherapy agents (22), and the established efficacy of GemOx in relapsed or refractory DLBCL (23). However, no objective response was achieved, likely attributable to immunophenotypic evolution toward a plasmablastic phenotype, intrinsic resistance of MYC/BCL2-coexpressing lymphomas to BTK inhibition (24), and lack of immune-based combinations. Given the intrinsic chemoresistance, novel immunotherapeutic strategies warrant consideration. For instance, bispecific antibodies (e.g., targeting CD38 or B-cell membrane antigen) could redirect T cells to eliminate plasmablasts (25), whereas CD19- or CD22-directed CAR-T cell therapy has shown promise in refractory PBL, as documented in recent case reports and early-phase clinical trials (26). These approaches may bypass the limitations of conventional chemotherapy and targeted agents, offering a potential avenue for improving outcomes in this aggressive disease.

## 4 Conclusion

This rare the DLBCL-to-PBL evolution in EBV-negative PBL in immunocompetent hosts highlights the challenges due to lymphoma heterogeneity and disease evolution. It highlights the need for multisite tissue sampling and molecular diagnostics to characterize the disease biology at diagnosis and at disease development. Despite multiagent chemotherapy, the patient became resistant to the treatment due to such high-grade evolutions and the double-expressor lymphoma phenotype and died soon.

However, this report has limitations. Although clonal relatedness was confirmed by PCR-based immunoglobulin gene rearrangement analysis, more detailed genomic studies, including whole-exome and transcriptomic sequencing, were not performed. Therefore, the mutational landscape, signaling pathway disturbances, and possible therapeutic targets remain undefined. To address these limitations, future studies should incorporate WES and single-cell RNA sequencing to comprehensively identify potential driver mutations (e.g., in MYC, PRDM1, or NF-κB pathway genes) and delineate the clonal architecture and tumor microenvironment interactions that underlie this rare evolution. Furthermore, as a single case report, its findings have limited generalizability. Therefore, additional cases and systematic studies will be required to characterize the complete molecular profile of the evolution from DLBCL to PBL and determine optimal treatment strategies.

## Data Availability

The original contributions presented in the study are included in the article/supplementary material. Further inquiries can be directed to the corresponding author.
